# Combined impact of lifestyle factors on mortality: prospective cohort study in US women

**DOI:** 10.1136/bmj.a1440

**Published:** 2008-09-16

**Authors:** Rob M van Dam, Tricia Li, Donna Spiegelman, Oscar H Franco, Frank B Hu

**Affiliations:** 1 Department of Nutrition, Harvard School of Public Health, 665 Huntington Ave, Boston, MA 02115, USA; 2 Channing Laboratory, Department of Medicine, Brigham and Women’s Hospital, and Harvard Medical School, Boston MA 02115; 3 Department of Epidemiology, Harvard School of Public Health; 4 Department of Biostatistics, Harvard School of Public Health; 5 Unilever Corporate Research, Sharnbrook, Bedfordshire MK44 1LQ

## Abstract

**Objective** To evaluate the impact of combinations of lifestyle factors on mortality in middle aged women.

**Design** Prospective cohort study.

**Setting** Nurses’ health study, United States.

**Participants** 77 782 women aged 34 to 59 years and free from cardiovascular disease and cancer in 1980.

**Main outcome measure** Relative risk of mortality during 24 years of follow-up in relation to five lifestyle factors (cigarette smoking, being overweight, taking little moderate to vigorous physical activity, no light to moderate alcohol intake, and low diet quality score).

**Results** 8882 deaths were documented, including 1790 from cardiovascular disease and 4527 from cancer. Each lifestyle factor independently and significantly predicted mortality. Relative risks for five compared with zero lifestyle risk factors were 3.26 (95% confidence interval 2.45 to 4.34) for cancer mortality, 8.17 (4.96 to 13.47) for cardiovascular mortality, and 4.31 (3.51 to 5.31) for all cause mortality. A total of 28% (25% to 31%) of deaths during follow-up could be attributed to smoking and 55% (47% to 62%) to the combination of smoking, being overweight, lack of physical activity, and a low diet quality. Additionally considering alcohol intake did not substantially change this estimate.

**Conclusions** These results indicate that adherence to lifestyle guidelines is associated with markedly lower mortality in middle aged women. Both efforts to eradicate cigarette smoking and those to stimulate regular physical activity and a healthy diet should be intensified.

## Introduction

Diet, physical activity, adiposity, alcohol consumption, and cigarette smoking have been associated with risk of chronic diseases including type 2 diabetes,^1^ ^2^ cardiovascular diseases,^3^ ^4^ ^5^ and various cancers.^6^ ^7^ However, to identify priorities for clinical and public health efforts, understanding the magnitude of effects of risk factors, individually and in combination, on overall health is fundamental. One way to assess the impact of lifestyle factors on overall health is to evaluate the effects on mortality, which allows comparison of the magnitude of effects of individual factors as well as estimation of combined effects.

The proportion of deaths that is attributable to lifestyle factors has been estimated by Mokdad and colleagues and in the global burden of disease study, using data on relative risks and the prevalence of risk factors from multiple sources.^8^ ^9^ As a result of this broad approach, the imprecision and potential biases affecting the results were less transparent and the analysis of lifestyle factors was less detailed than can be achieved in a well characterised prospective cohort study. In a cohort study in 11 European countries, an estimated 60% of deaths from all causes during 10 years of follow-up could be attributed to lack of adherence to non-smoking, a healthy diet, regular physical activity, and moderate alcohol intake.^10^ However, this study included only 2339 participants, who were elderly and mostly male, and whether the findings apply to younger populations and women thus remains unclear. We therefore examined combinations of lifestyle factors in relation to cancer, cardiovascular, and all cause mortality during 24 years of follow-up among middle aged women who participated in the nurses’ health study. We also estimated population attributable risks, the proportion of deaths during follow-up that could potentially have been avoided by adherence to lifestyle guidelines.

## Methods

### Study population

The nurses’ health study is a prospective cohort study that was established in 1976 when 121 700 female registered US nurses, aged 30 to 55 years, completed a mailed questionnaire on known and suspected risk factors for chronic diseases. Since then, participants have been sent biennial follow-up questionnaires to update information on lifestyle and health conditions. For the current analysis, we began follow-up in 1980, because this was the first year when diet was assessed. In 1980, 98 462 women, aged 34 to 59 years, completed the questionnaire. We excluded women with previously diagnosed cancer (except non-melanoma skin cancer) or cardiovascular disease (n=8527). We also excluded alcohol abstainers who reported on the 1980 questionnaire a great decrease in alcohol consumption during the previous decade (n=2797), because this could be subsequent to alcoholism or disease. Similarly, we excluded women with a body mass index below 18.5 (n=1848), because this may be due to weight loss secondary to preclinical diseases.^11^ Finally, we excluded participants who left 10 or more items blank on the 1980 diet questionnaire, those with implausibly high or low reported energy intakes (below 2.09 or above 14.63 MJ/day), those with ages outside the eligible range, and those with missing information for smoking or body mass index (n=7562). After exclusions, a total of 77 782 women remained for the current analysis. Completion of the questionnaire was considered to imply informed consent.

### Assessment of risk factors

Diet was assessed with a 61 item food frequency questionnaire in 1980.^12^ An expanded questionnaire including approximately 120 food items was used to update information about diet every two to four years. Participants were asked how often, on average, during the previous year they had consumed a specified common unit or portion size of a specific type of food. The participants could choose from nine response categories ranging from “never or less than once a month” to “six or more times a day.” Nutrient intakes were calculated by summing the nutrient content of a unit of each food multiplied by a weight proportional to the frequency of its use. Validation studies in this cohort using biochemical markers and diet records as references indicated that the food frequency questionnaire estimated dietary intakes with reasonably good accuracy.^12^ ^13^ ^14^

Information on disease history and cigarette smoking was assessed on each biennial questionnaire. Frequency of physical activity during the previous year was assessed in 1980, and this information was updated every two to four years.^15^ Height (in inches) was assessed in the 1976 questionnaire, weight (in pounds) was assessed on the 1980 questionnaire, and the body mass index was calculated as weight in kilograms divided by the square of height in metres. In our validation study, the correlation between self reported and technician measured weight was 0.97.^16^ Father’s occupation when the participant was 16 years of age was assessed on the 1976 questionnaire. In 1992, we also asked about the degrees the participant had received (registered nurse, bachelors, masters, doctorate) and, for women who were married or widowed, the highest level of education that their husband completed (less than high school, some high school, high school graduate, college graduate, graduate school).

### Classification of low risk categories

For adiposity, we defined low risk as a body mass index between 18.5 and 25.0.^17^ For physical activity, we defined low risk as an average of at least 30 minutes a day of activity of at least moderate intensity (requiring ≥3 metabolic equivalents an hour, including brisk walking), consistent with existing guidelines.^18^ For cigarette smoking, we defined low risk as never smoking. For alcohol consumption, we defined low risk as light to moderate consumption (≥1 and <15 g/day—that is, up to approximately one drink a day).

To quantify the healthiness of the diet, we used the previously designed alternative healthy eating score.^19^ For this analysis, we decided a priori to use only seven of the nine original items: we did not include multivitamin intake, because this has become less relevant as a folate source as a result of widespread fortification of foods, and we did not include alcohol intake, because we wanted to present results on alcohol intake separately. For each of the remaining seven items, we assigned a score between 0 (least healthy) and 10 (recommended intake). We then added the scores for the individual items, resulting in a minimum healthy diet score of 0 and a maximum healthy diet score of 70. The seven included items were vegetables (recommended: at least five servings a day), fruit (at least four servings a day), nuts and soya (at least one serving a day), ratio of fish and poultry to red meat (ratio at least four or red meat less than twice a month), cereal fibre (≥15 g/day), *trans*-fat (≤0.5% of total energy), and the polyunsaturated to saturated fatty acid ratio (ratio at least one).^19^ We considered participants with a healthy diet score in the upper two fifths (highest 40%) to be in the low risk category for diet.

### Ascertainment of mortality

Deaths were reported by next of kin, the postal authorities, or both or were ascertained through searching for non-responders in the National Death Index. Follow-up for deaths in the National Death Index has been estimated to be 98% complete for this cohort.^20^ Information on the cause of death was obtained from the next of kin (for external deaths such as those due to traffic accidents), death certificates, or medical records. We requested permission from the next of kin to review medical records for suspected deaths due to cancer or cardiovascular diseases if we did not yet have these records for follow-up of disease incidence. The cause of death was determined after review by physicians and primarily based on medical records if both medical records and death certificates were available. We used ICD-8 (international classification of diseases, 8th revision) codes to distinguish deaths due to cancer (140-207) and cardiovascular diseases (390-459 and 795).

### Statistical analysis

Women contributed follow-up time from the date of return of the baseline questionnaire to the date of death or 1 June 2004, whichever came first. We used pooled logistic regression analysis stratified by two year calendar time periods to estimate multivariate relative risks and corresponding 95% confidence intervals. We chose this method because it obtains all information needed for the calculation of partially adjusted population attributable risk estimates, including estimation of the relative risks for age.^21^ Pooled logistic regression analysis is asymptotically equivalent to Cox proportional hazards regression if time intervals are relatively short and the probability of the outcome in the intervals is small,^22^ conditions that were satisfied for this analysis.

We calculated population attributable risks, which are estimates of the percentage of deaths during follow-up that would not have occurred if all women had been in the low risk category for lifestyle factors, assuming that the observed associations represent causal effects. For these analyses, we compared women in the low risk category for each of the component risk factors with all other women.^23^ We calculated population attributable risks by using previously described formulas that were elaborated for this specific study design and are appropriate for use with multivariate adjusted relative risks (SAS macro: www.hsph.harvard.edu/faculty/spiegelman/software.html).^21^ ^24^ ^25^ Because of the large computational computer capacity needed for this calculation, we reduced the number of categories for the covariable calendar time by combining 12 two year time periods into four periods. We estimated prevalence of exposures for 1990, because this year is midway during follow-up and approximates average exposure during follow-up.

To estimate relative risks for cigarette smoking and diet, we updated information during follow-up by using the most recently available information. For physical activity and alcohol intake, we used the cumulative average from all available questionnaires up to the start of each two year follow-up.^26^ To minimise bias owing to the effects of poor health before death leading to weight loss,^11^ we used the baseline (1980) body mass index in our analysis. In a sensitivity analysis, we examined potential confounding by measures of socioeconomic status by adding father’s occupation, education degree received by the participant, and education level of the husband to the multivariate model. Because education level was only assessed in 1992, we did this analysis for follow-up from 1992 until 2004.

We considered two tailed P values lower than 0.05 to be statistically significant. We used SAS software, version 9.1 for all analyses.

## Results

During 1 759 408 person years of follow-up we documented 8882 deaths, including 1790 from cardiovascular disease and 4527 from cancer. Table 1 shows the multivariate adjusted relative risks for lifestyle factors and death during follow-up. Cigarette smoking, higher body mass index, less physical activity, and a lower healthy diet score were all associated with increased cardiovascular, cancer, and all cause mortality. Alcohol consumption was associated with a lower risk of cardiovascular mortality than alcohol abstinence. However, heavy alcohol consumption was associated with an increased risk of cancer mortality. As a result, light to moderate alcohol consumption was associated with the lowest all cause mortality.

**Table 1 tbl1:** Multivariate relative risk of death from any cause, cardiovascular disease, and cancer during 24 years of follow-up according to body mass index, cigarette smoking, alcohol consumption, physical activity, and diet*

	Person years	Deaths from any cause			Cardiovascular deaths			Cancer deaths	
		Cases	Relative risk (95% CI)		Cases	Relative risk (95% CI)		Cases	Relative risk (95% CI)
**Body mass index (kg/m^2^)**									
18.5-24.9	1 174 514	5095	1.00 (1.00 to 1.00)		855	1.00 (1.00 to 1.00)		2747	1.00 (1.00 to 1.00)
25-29.9	404 856	2359	1.18 (1.12 to 1.24)		511	1.46 (1.31 to 1.63)		1195	1.14 (1.06 to 1.22)
≥30.0	180 039	1428	1.67 (1.57 to 1.78)		424	2.81 (2.49 to 3.17)		585	1.32 (1.21 to 1.45)
**Cigarette smoking**									
Never	787 104	2998	1.00 (1.00 to 1.00)		595	1.00 (1.00 to 1.00)		1519	1.00 (1.00 to 1.00)
Past	667 250	4076	1.52 (1.44 to 1.59)		743	1.49 (1.33 to 1.66)		2079	1.47 (1.37 to 1.57)
Current 1-14/day	104 428	609	1.94 (1.77 to 2.12)		145	2.61 (2.17 to 3.14)		318	1.82 (1.61 to 2.06)
Current ≥15/day	200 626	1199	2.32 (2.16 to 2.49)		307	3.34 (2.88 to 3.87)		611	2.10 (1.90 to 2.32)
**Alcohol consumption† (g/day)**									
0	386 395	2044	1.00 (1.00 to 1.00)		502	1.00 (1.00 to 1.00)		887	1.00 (1.00 to 1.00)
1-4	745 039	3589	0.81 (0.76 to 0.85)		712	0.69 (0.61 to 0.77)		1878	0.97 (0.90 to 1.06)
5-14	405 344	1861	0.80 (0.75 to 0.86)		324	0.63 (0.54 to 0.73)		1023	0.99 (0.90 to 1.09)
15-29	155 024	900	0.90 (0.83 to 0.98)		168	0.75 (0.62 to 0.90)		492	1.11 (0.99 to 1.24)
≥30	67 369	485	1.11 (1.00 to 1.23)		82	0.75 (0.59 to 0.96)		246	1.26 (1.09 to 1.46)
**Physical activity‡ (hours/week)**									
0-0.4	116 915	1143	1.00 (1.00 to 1.00)		231	1.00 (1.00 to 1.00)		513	1.00 (1.00 to 1.00)
0.5-1.9	596 872	3366	0.84 (0.78 to 0.90)		715	0.94 (0.81 to 1.09)		1644	0.88 (0.79 to 0.97)
2.0-3.4	370 631	1690	0.77 (0.71 to 0.83)		345	0.87 (0.73 to 1.03)		896	0.83 (0.75 to 0.93)
3.5-5.4	201 459	768	0.72 (0.65 to 0.79)		126	0.70 (0.56 to 0.87)		446	0.82 (0.72 to 0.94)
≥5.5	248 639	636	0.63 (0.57 to 0.69)		96	0.57 (0.45 to 0.73)		379	0.73 (0.64 to 0.84)
**Healthy diet score§**									
Fifth 1	337 747	2122	1.00 (1.00 to 1.00)		443	1.00 (1.00 to 1.00)		1038	1.00 (1.00 to 1.00)
Fifth 2	348 559	1848	0.85 (0.79 to 0.90)		427	0.96 (0.84 to 1.10)		891	0.83 (0.76 to 0.91)
Fifth 3	355 705	1766	0.80 (0.75 to 0.85)		344	0.78 (0.67 to 0.90)		895	0.81 (0.74 to 0.89)
Fifth 4	359 442	1701	0.76 (0.71 to 0.81)		326	0.75 (0.65 to 0.87)		894	0.80 (0.73 to 0.87)
Fifth 5	357 955	1445	0.65 (0.61 to 0.70)		250	0.59 (0.51 to 0.70)		809	0.72 (0.65 to 0.79)

*Relative risks adjusted for age (5 year categories), time period (12 periods), and other risk factors included in table.

†Three deaths and 239 person years not included because of missing values.

‡Of moderate to vigorous intensity; 1279 deaths and 224 893 person years not included because of missing values.

§In 1990 healthy diet score was <22.61 (median 18.65) for fifth 1, 22.61-28.85 (median 25.89) for fifth 2, 28.86-34.57 (median 31.68) for fifth 3, 34.58-41.38 (median 37.72) for fifth 4, and >41.39 (median 46.34) for fifth 5.

Table 2 shows the multivariate adjusted relative risks and population attributable risks of mortality for the high risk compared with the low risk category of lifestyle factors. The estimated population attributable risks were 28% for cigarette smoking, 14% for being overweight, 17% for lack of physical activity, 13% for low diet quality, and 7% for not having light to moderate alcohol consumption. Population attributable risks were higher for cardiovascular mortality than for cancer mortality. Among never smokers, the relative risk of mortality for being overweight was higher than that for the whole study population (1.55, 95% confidence interval 1.44 to 1.66), resulting in a higher population attributable risk (22%, 18% to 27%).

**Table 2 tbl2:** Relative risk and population attributable risk (PAR) (95% confidence intervals) of all cause, cardiovascular, and cancer mortality during 24 years of follow-up*

Variable	Women in high risk category (%)†	Death from any cause			Cardiovascular death			Cancer death	
		Relative risk	PAR (%)		Relative risk	PAR (%)		Relative risk	PAR (%)
Ever *v* never smoking	56	1.66 (1.59 to 1.74)	27.9 (24.6 to 31.2)		1.83 (1.65 to 2.02)	32.7 (25.7 to 39.4)		1.59 (1.49 to 1.70)	26.8 (21.3 to 31.3)
Body mass index ≥25 *v* 18.5-24.9	48	1.32 (1.27 to 1.38)	14.2 (11.6 to 16.9)		1.85 (1.68 to 2.04)	30.6 (25.5 to 35.4)		1.19 (1.12 to 1.27)	8.3 (4.4 to 12.2)
Physical activity <30 min/day *v* ≥30 min/day	75	1.25 (1.17 to 1.32)	16.5 (11.7 to 21.1)		1.47 (1.27 to 1.69)	27.7 (17.2 to 37.5)		1.13 (1.05 to 1.22)	9.3 (2.7 to 15.9)
Healthy diet score in lower three fifths *v* upper two fifths	59	1.25 (1.19 to 1.30)	12.9 (9.6 to 16.2)		1.35 (1.22 to 1.50)	17.7 (10.3 to 25)		1.16 (1.09 to 1.24)	8.8 (4.1 to 13.4)
Heavier drinking or abstaining‡ *v* light to moderate alcohol intake	34	1.18 (1.13 to 1.24)	7.4 (4.6 to 10.2)		1.31 (1.19 to 1.44)	11.3 (4.9 to 17.5)		1.06 (1.00 to 1.12)	3.1 (0.8 to 5.3)

*Relative risks and population attributable risks adjusted for age (5 year categories), time period (four periods), and other risk factors included in table.

†Based on prevalence in 1990.

‡0 or ≥15 g/day alcohol.

We also evaluated combinations of lifestyle risk factors in relation to mortality. As shown in the figure[Fig fig1], cardiovascular, cancer, and all cause mortality increased with an increasing number of risk factors. The relative risk for combining cigarette smoking, being overweight, lack of physical activity, and a low healthy diet score compared with none of the risk factors was 6.91 (4.50 to 10.63) for cardiovascular mortality, 2.65 (2.14 to 3.28) for cancer mortality, and 3.41 (2.90 to 4.00) for all cause mortality (table 3). The population attributable risk for having any of these four risk factors was 72% for cardiovascular mortality, 44% for cancer mortality, and 55% for all cause mortality. When we also considered alcohol consumption, the population attributable risks for having any of the five lifestyle risk factors were modestly greater than for the four risk factors (table 3).

**Figure fig1:**
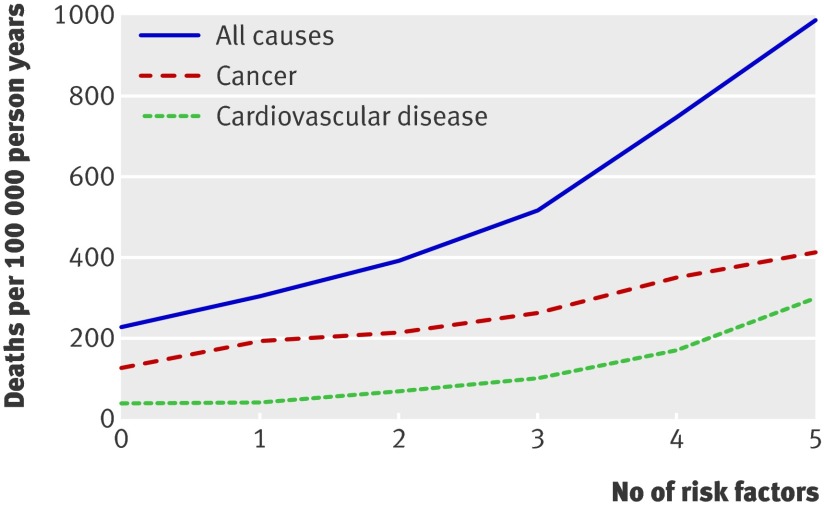
Age standardised all cause, cancer, and cardiovascular mortality during 24 years of follow-up by number of lifestyle risk factors. Lifestyle risk factors included cigarette smoking (ever), lack of physical activity (<30 min/day moderate to vigorous intensity activity), low diet quality (lowest three fifths of healthy diet score), alcohol intake of 0 or ≥15 g/day, and overweight (body mass index ≥25)

**Table 3 tbl3:** Risk of mortality during 24 years of follow-up according to combinations of lifestyle risk factors*

	Death from any cause	Cardiovascular death	Cancer death
**Four risk factors: smoking, overweight, low diet quality, low physical activity†**			
Relative risk (95% CI):			
No risk factors (3.4%)‡	1.00	1.00	1.00
One risk factor (16%)	1.30 (1.10 to 1.53)	1.43 (0.91 to 2.24)	1.32 (1.06 to 1.63)
Two risk factors (33%)	1.75 (1.49 to 2.05)	2.42 (1.58 to 3.71)	1.61 (1.231 to 1.98)
Three risk factors (34%)	2.52 (2.15 to 2.95)	3.98 (2.60 to 6.08)	2.12 (1.73 to 2.60)
Four risk factors (13%)	3.41 (2.90 to 4.00)	6.91 (4.50 to 10.63)	2.65 (2.14 to 3.28)
Population attributable risk (%) for having any of the four risk factors (95% CI)	54.8 (46.7 to 61.9)	72.0 (58.6 to 81.6)	44.3 (31.2 to 55.8)
**Five risk factors: above four and alcohol abstinence or heavy drinking§**			
Relative risk (95% CI):			
No risk factors (2.4%)‡	1.00	1.00	1.00
One risk factor (12%)	1.34 (1.09 to 1.64)	1.13 (0.67 to 1.89)	1.55 (1.18 to 2.02)
Two risk factors (27%)	1.70 (1.40 to 2.07)	1.88 (1.15 to 3.05)	1.71 (1.32 to 2.22)
Three risk factors (34%)	2.25 (1.85 to 2.72)	2.80 (1.73 to 4.54)	2.07 (1.60 to 2.68)
Four risk factors (21%)	3.27 (2.70 to 3.97)	4.77 (2.94 to 7.72)	2.79 (2.15 to 3.62)
Five risk factors (4.2%)	4.31 (3.51 to 5.31)	8.17 (4.96 to 13.47)	3.26 (2.45 to 4.34)
Population attributable risk (%) for having any of the five risk factors (95% CI)	58.1 (49.3 to 65.7)	75.2 (60.9 to 84.7)	46.0 (31.7 to 58.3)

*Relative risks and population attributable risks adjusted for age (5 year age categories) and time period (four periods); additionally adjusted for alcohol consumption (0, 1-4, 5-14, 15-29, ≥30 g/d) for “four risk factor” model.

†Overweight: body mass index ≥25; low diet quality: healthy diet score in lower three fifths; low physical activity: <30 min/day.

‡Prevalence in 1990.

§0 or ≥15 g/day alcohol.

Results among never smokers were consistent with those for the whole study population (table 4). We also examined whether results differed for younger (<60 years) and older (≥60 years) participants by recalculating the relative risks for these subgroups and using the prevalence of risk factor specific for these subgroups. The population attributable risk for all cause mortality for the five risk factors combined (smoking, diet, physical activity, overweight, alcohol consumption) was 51% (30% to 67%) for younger women and 63% (52% to 72%) for older women. Finally, we examined potential confounding by measures of socioeconomic status (father’s occupation, education level of participant and husband). Adjustment for these variables did not materially alter the association between any of the lifestyle factors and mortality during follow-up (data not shown).

**Table 4 tbl4:** Risk of mortality during 24 years of follow-up according to number of lifestyle risk factors in never smokers*

	Death from any cause	Cardiovascular death	Cancer death
**Three risk factors: low diet quality, low physical activity, overweight†**			
Relative risk (95% CI):			
No risk factors (7.7%)‡	1.00	1.00	1.00
One risk factor (27%)	1.20 (1.02 to 1.43)	1.44 (0.91 to 2.27)	1.19 (0.95 to 1.49)
Two risk factors (41%)	1.54 (1.31 to 1.81)	2.24 (1.44 to 3.46)	1.37 (1.11 to 1.70)
Three risk factors (25%)	2.17 (1.83 to 2.57)	3.58 (2.30 to 5.58)	1.75 (1.39 to 2.19)
Population attributable risk (%) for having any of the three risk factors (95% CI)	34.3 (18.9 to 48.0)	56.1 (27.7 to 75.5)	22.8 (−0.01 to 44.3)
**Four risk factors: above three and alcohol abstinence or heavy drinking§**			
Relative risk (95% CI)			
No risk factors (5.4%)‡	1.00	1.00	1.00
One risk factor (20%)	1.25 (1.01 to 1.53)	1.18 (0.70 to 1.99)	1.39 (1.05 to 1.83)
Two risk factors (34%)	1.48 (1.22 to 1.81)	1.78 (1.08 to 2.92)	1.42 (1.08 to 1.85)
Three risk factors (30%)	1.95 (1.60 to 2.38)	2.64 (1.61 to 4.33)	1.70 (1.30 to 2.22)
Four risk factors (10%)	2.64 (2.13 to 3.26)	3.94 (2.35 to 6.60)	2.11 (1.57 to 2.83)
Population attributable risk (%) for having any of the four risk factors (95% CI)	41.5 (24.3 to 56.2)	60.9 (28.3 to 80.9)	25.7 (−1.4 to 49.3)

*Relative risks and population attributable risks adjusted for age (5 year age categories) and time period (four periods); additionally adjusted for alcohol consumption (0, 1-4, 5-14, 15-29, ≥30.0 g/d) for the “three risk factor” model.

†Low diet quality: healthy diet score in lower three fifths; low physical activity: <30 min/day; overweight: body mass index ≥25.

‡Prevalence in 1990.

§0 or ≥15 g/day alcohol.

## Discussion

In this study of 77 782 middle aged US women, never smoking, engaging in regular physical activity, eating a healthy diet, and avoiding becoming overweight were each associated with a markedly lower mortality during 24 years of follow-up. We estimated that 55% of all cause mortality, 44% of cancer mortality, and 72% of cardiovascular mortality during follow-up could have been avoided by adherence to these four lifestyle guidelines. Light to moderate alcohol consumption (up to one drink a day) was also associated with a lower risk of all cause mortality during follow-up.

### Results in relation to other studies

Smoking, overweight, physical activity, and quality of diet have consistently been associated with risk of chronic diseases and mortality in prospective cohort studies.^6^ ^7^ ^10^ ^11^ ^18^ ^19^ ^27^ Not all of these risk factors have been studied in randomised controlled trials with disease incidence or mortality end points, and this may never be done for feasibility or ethical reasons. However, findings from randomised controlled trials support the protective effect of a prudent Mediterranean-style diet and substitution of polyunsaturated for saturated fat for coronary heart disease^3^ ^4^; of the combination of physical activity, a healthy diet, and moderate weight loss for type 2 diabetes^2^; and of smoking cessation for premature mortality.^28^ In addition, randomised controlled trials have shown beneficial effects of moderate alcohol consumption, reduced *trans*-fat intake, high fruit and vegetable intake, and whole grain intake on biological markers of cardiovascular risk.^29^ ^30^ ^31^ ^32^

Few previous studies have examined combinations of lifestyle factors in relation to mortality. In the EPIC-Norfolk study, lifestyle factors were studied in relation to mortality in men and women aged 45 to 79 years during an average of 11 years of follow-up. Participants who smoked, were physically inactive, were non-moderate alcohol consumers, and had low fruit and vegetable intakes (measured by plasma vitamin C concentrations) had a relative risk of all cause mortality during follow-up of 4.04 (2.95 to 5.54) compared with participants who met none of these criteria.^27^ In another British prospective study, the estimated probability of surviving 15 years free of cardiovascular events and diabetes for a man aged 50 years ranged from 89% in a moderately physically active man with a body mass index between 20 and 24 who had never smoked to 42% in an inactive smoker with a body mass index of 30 or higher.^33^ In a cohort of elderly Europeans aged 70 to 90 years, the combination of non-smoking, a Mediterranean-style diet, regular physical activity, and moderate alcohol consumption was associated with a relative risk of 10 year all cause mortality of 0.35 (0.28 to 0.44) compared with adherence to only one or fewer of these lifestyle factors.^10^ The estimated population attributable risk for the combination of lifestyle factors was 60% for all cause mortality, 61% for cardiovascular mortality, and 60% for cancer mortality. For women in high income countries in the global burden of disease study, the estimated population attributable risk for mortality was 14% for smoking, 8% for overweight, 5% for physical inactivity, 4% for low fruit and vegetable intake, and −3% for alcohol use.^9^ Mokdad and colleagues estimated that the population attributable risks for US mortality in 2000 was 18% for tobacco, 15% for poor diet and physical inactivity, and 3.5% for excess alcohol consumption.^8^ Thus, our results are consistent with those from previous cohort studies that included European and male participants and suggest that the population attributable risk estimates from studies using an indirect approach may be conservative for the demographic group that we studied.

Heavy alcohol consumption was associated with higher cancer mortality in our study and has been associated with an increased risk of various cancers, liver cirrhosis, hypertension, psychiatric disorders, injuries, and violence in other studies.^34^ ^35^ Consistent with previous studies,^10^ ^36^ we found that light to moderate alcohol consumption was associated with lower cardiovascular mortality. However, even moderate alcohol consumption has been associated with a higher risk of breast cancer and traffic accidents.^35^ ^37^ In addition, people with moderate alcohol consumption may be more likely to transition to heavy consumption, and a greater average alcohol consumption may facilitate heavy consumption in communities.^38^ For individual people, the balance of risks and benefits of moderate alcohol consumption may depend on other characteristics; possible benefits may exist for older women with cardiovascular risk factors,^39^ and a greater likelihood of adverse effects may exist for women with a personal or family history of alcoholism, alcohol related cancers, or risk factors for these conditions.^35^ ^37^ Given the complexity of the effects of alcohol consumption on personal health, women could usefully discuss these risks and benefits with their physician.

### Strengths and limitations

Strengths of our study include the large sample size, the prospective design with 24 years of follow-up and high response rates, and the repeated collection of detailed information on lifestyle. Some measurement error is inevitable, particularly in the assessment of diet and physical activity, and is likely to have weakened the associations seen. We would probably have seen a lower mortality if we had used more restrictive criteria for the low risk group (for example, healthy diet score in the highest fifth). However, our results indicate that even modest differences in lifestyle can have a substantial impact on reducing mortality. Although residual confounding can never be excluded in non-randomised studies, evidence from prospective studies in other populations and randomised controlled trials supports our findings. Because incomplete adjustment for smoking habits can weaken the association between overweight and mortality,^11^ our estimates for the association between overweight and mortality in never smokers may be more accurate than those for the total study population. Variation in socioeconomic status in our study of registered nurses was more limited than in the general population. Consistent with results from a British study,^33^ adjustment for various measures of socioeconomic status did not appreciably affect associations between lifestyle factors and mortality during follow-up. In analyses with mortality as an end point, confounding by poor health that precedes death and affects lifestyle habits (for example, resulting in weight loss, reduced alcohol consumption, and physical inactivity) is of particular concern. For this reason, we excluded women with conditions that may reflect this poor health status at baseline. Also, our findings were consistent with results in analyses of lifestyle factors and risk of incidence of chronic diseases that are less likely to be affected by this type of confounding.^1^ ^5^ ^37^ ^40^

Because population attributable risks depend on both relative risks and the prevalence of risk factors (the higher the prevalence, the higher the population attributable risk) the prevalence of risk factors has to be considered when generalising our findings to other populations. For example, compared with the prevalence of 48% in our cohort, overweight (body mass index ≥25) was more common in women in national surveys in the United States (62%) and England (59%), similar in Greece (46%) and Spain (48%), and less common in Italy (35%) and the Netherlands (39%).^41^ ^42^ As a result, population attributable risks for individual risk factors will differ somewhat by population, but we believe that our overall conclusions are generally applicable to middle aged women in high income countries. However, our participants were predominantly white and confirmation in other ethnic groups is warranted.

### Conclusions

Avoiding cigarette smoking is of pivotal importance for the prevention of premature death. In our study of middle aged women, adherence to lifestyle guidelines involving a healthy diet, regular physical activity, and weight management was also associated with markedly lower mortality. Of note, our results indicate that a healthy diet and regular physical activity have important health benefits independent of reducing adiposity. These findings underscore the importance of intensifying both efforts to eradicate cigarette smoking and those aimed at improving diet and physical activity.

**What is already known on this topic**

Many studies have shown that individual lifestyle factors are associated with risk of chronic diseases
Few studies have evaluated the effects of combinations of lifestyle factors on mortality

**What this study adds**

Most deaths during 24 years of follow-up in middle aged women could have been avoided by a combination of not smoking, maintaining a healthy weight, regular physical activity, and a healthy diet
These findings underscore the importance of intensifying both efforts to eradicate cigarette smoking and those aimed at improving diet and physical activity

## Notes

We thank the participants of the nurses’ health study for their continued participation, Ellen Hertzmark for expert programming help, and Walter Willett and Meir Stampfer for their valuable comments on the manuscript. The article adheres to the STROBE guidelines (www.strobe-statement.org/).

Ethical approval: The institutional review board at Brigham and Women’s Hospital approved this study.

Provenance and peer review: Not commissioned; externally peer reviewed.

## Notes

**Cite this as:** *BMJ* 2008;337:a1440
